# Complete Remission from Salvage Volumetric Modulated Arc Therapy in Recurrent Adenocarcinoma of the Jejunum with Retroperitoneal Metastasis Eleven Years after Diagnosis: A Rare Case of Cure

**DOI:** 10.7759/cureus.11457

**Published:** 2020-11-12

**Authors:** Ming Pan

**Affiliations:** 1 Radiation Oncology, Windsor Regional Hospital Cancer Program, Windsor, CAN

**Keywords:** small bowel cancer, adenocarcinoma, retroperitoneal metastasis, volumetric modulated arc therapy, concurrent chemoradiation, complete remission

## Abstract

Small bowel adenocarcinoma (SBA) is rare and carries very poor prognosis if there is metastasis. This case shows the benefit of offering aggressive chemoradiation for recurrent adenocarcinoma of the jejunum with retroperitoneal metastasis to achieve the best quality of life (QoL) and potential cure. A complete response (CR) was observed following volumetric modulated arc therapy (VMAT) to biopsy-proven retroperitoneal lymph node metastasis four years after the initial diagnosis of stage pT4pN1 adenocarcinoma of the jejunum. Retroperitoneal lymph node dissection was not performed due to the excellent local control. There was no residual cancer or further metastasis seen on follow-up computed tomography (CT) imaging. The patient remains cancer free and asymptomatic eleven years after the initial diagnosis.

## Introduction

Small bowel cancer (SBC) is a rare disease. It only represents 0.6% of all new cancer cases in the USA and accounts for 0.3% of all cancer death in 2020 [1]. Different histological types of SBC include small bowel adenocarcinoma (SBA), neuroendocrine tumor, sarcoma, gastrointestinal stromal tumor (GIST), and lymphoma. SBA accounts for 30%-50% of all SBCs with duodenum being the main site of occurrence. Metastatic SBA of the jejunum is rarely reported and carries very poor outcome [2]. The treatment is usually palliative chemotherapy [3-6]. Targeted therapy, against the epidermal growth factor receptor (EGFR) pathway or the angiogenic pathway, is not yet established in any guidelines despite the over-expression of EGFR or vascular epithelial growth factor (VEGF)-A observed in SBA [7].

We report a case of recurrent biopsy-proven retroperitoneal lymph node metastasis four years after the surgery for stage pT4 pN1 adenocarcinoma of the jejunum. Retroperitoneal lymph node dissection was not performed due to the excellent local control with aggressive chemoradiation using volumetric modulated arc therapy (VMAT). There were no significant toxicities, no residual cancer, or further metastasis. The patient was cured and maintained good quality of life (QoL) eleven years after the initial diagnosis.

## Case presentation

**Figure 1:**
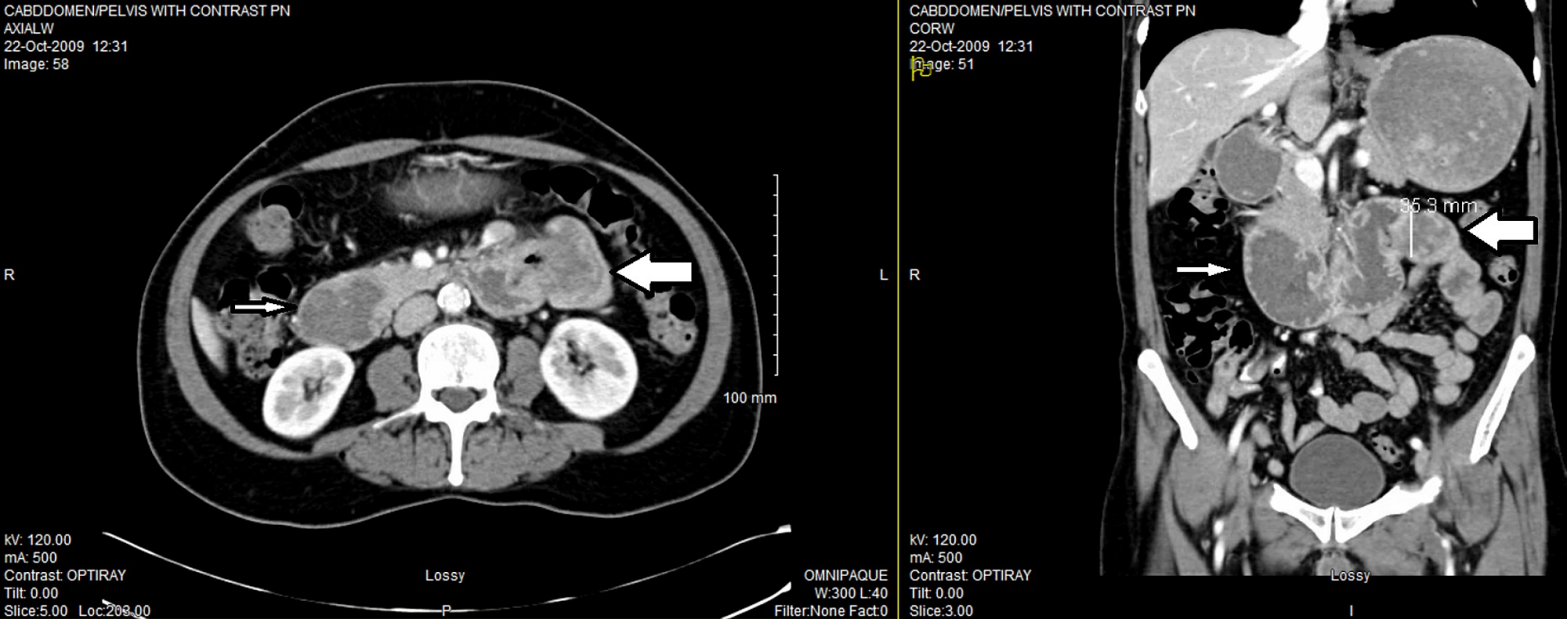
CT scan of the abdomen and pelvis in October 2009 showing a polypoid mass of the proximal jejunum (big arrow), incomplete bowel obstruction, marked dilatation of the stomach, and the duodenum (small arrow).

A 66-year-old non-smoker, non-drinker male presented with weight loss, anemia, nausea and vomiting large amount of bile. Colonoscopy was largely normal. Esophago-gastro-duodenoscopy reviewed tumors in jejunum. Computed tomography (CT) imaging showed a polypoid mass with mural thickening of the proximal jejunum, slightly distal to the ligament of Treitz, measuring 4.6 x 5.3 x 3.5 cm with incomplete bowel obstruction causing marked dilatation of the duodenum and the stomach with retained food products. Slight increased stranding is identified in the adjacent mesentery with mesenteric lymph nodes up to 0.6 cm in the short axis diameter indicating possible contiguous spread (Figure 1).

He had surgery in November 2009 for a stage pT4b pN1 adenocarcinoma of the jejunum measuring 6 x 3.5 x 0.8 cm in size. It was low grade, ulcerating and infiltrative, with clear margins but three metastatic mesenteric lymph nodes out of the 14 removed. There was lympho-vascular invasion and perineural invasion present. The tumour involved the serosa and visceral peritoneum. He underwent adjuvant chemotherapy in the form of oral capecitabine, 1000 mg/m^2^ twice a day for 14 days and then one week off as one cycle. He completed six cycles of chemotherapy during four and a half months.

**Figure 2:**
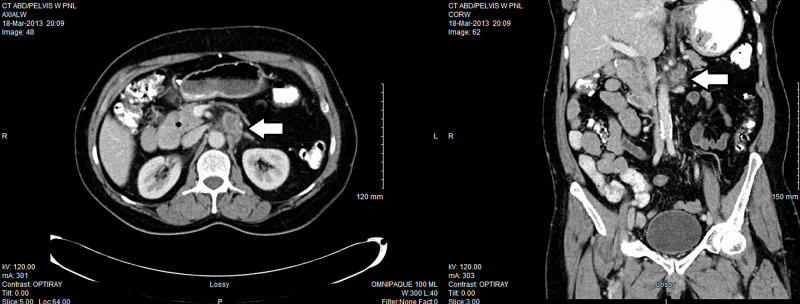
CT scan in March 2013 showing a new large retroperitoneal lymph node (white arrow) abutting the aorta, SMA and left renal vein. SMA, superior mesentery artery

**Figure 3:**
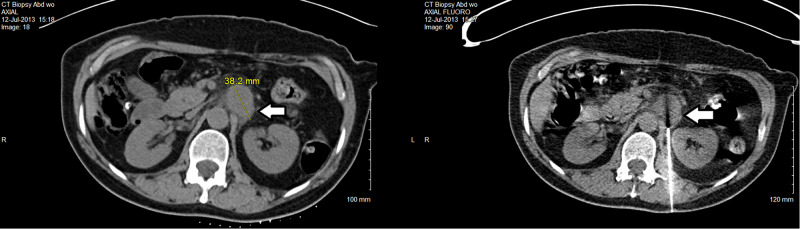
CT-guided core biopsy in July 2013 confirmed recurrent moderately differentiated adenocarcinoma (white arrow).

His tumour marker carcinoembryonic antigen (CEA) had been always normal, ranging between 1.2 and 2.9 µg/L. But he was noted to have a new large retroperitoneal lymph node measuring 4.3 x 3.5 cm on CT scan in March 2013 (Figure 2). CT-guided core biopsy confirmed recurrent moderately differentiated adenocarcinoma with extensive tumour necrosis favouring metastatic adenocarcinoma from the jejunum (Figure 3). Immunohistochemistry was similar to the original pathological specimen in 2009, i.e. positive for cytokeratin 7, villin and CDX-2, negative for prostate-specific antigen (PSA), prostatic acid phosphatase (PAP), and cytokeratin 20.

**Figure 4:**
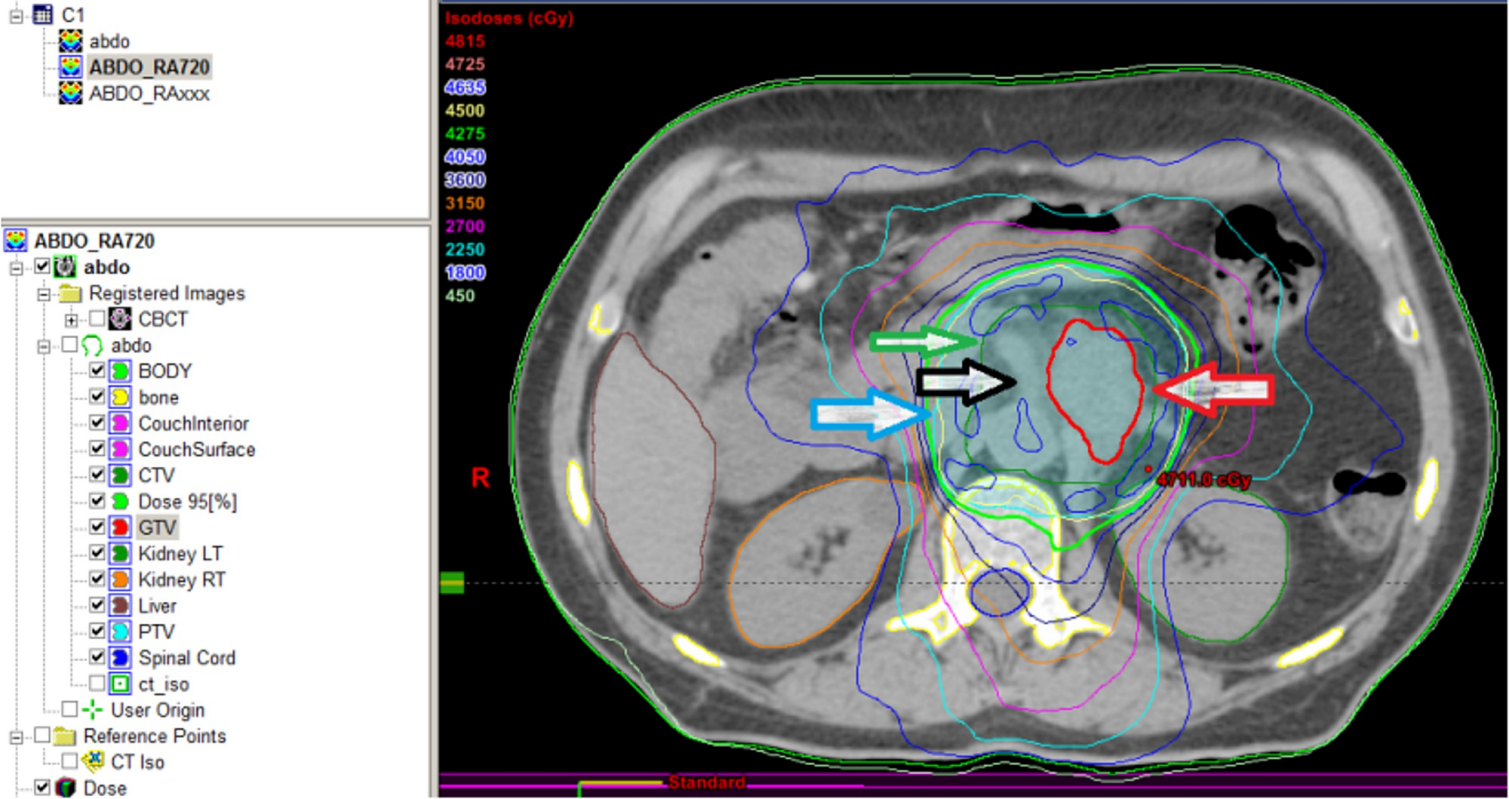
VMAT plan showing different dose curves covering the GTV (red arrow), CTV (green arrow), and PTV (blue arrow), while avoiding high dose in the OARs. Note the location of SMA (black hollow arrow). VMAT, volumetric modulated arc therapy; GTV, gross tumor volume; CTV, clinical target volume; PTV, planning target volume; OARs, organs at risk; SMA, superior mesentery artery

**Figure 5:**
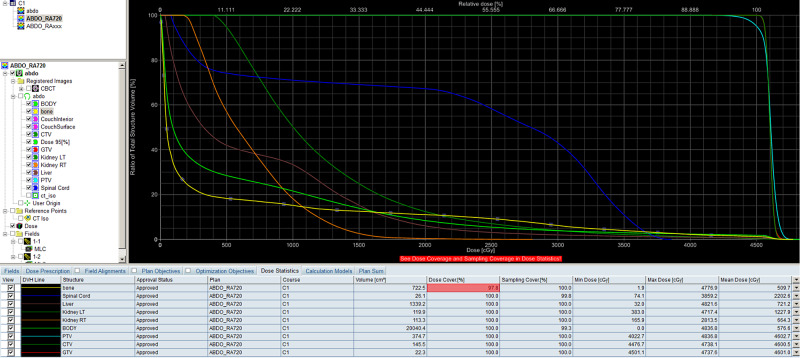
DVH showing all OAR tolerance dose constraints were met in VMAT plan. DVH, dose volume histogram; OAR, organs at risk; VMAT, volumetric modulated arc therapy

His case was discussed in our Multidisciplinary Tumor Board Meeting. This solitary metastasis could not be safely surgically removed due to the critical organs nearby, including the aorta, superior mesentery artery (SMA), left renal artery, and vein. Stereotactic body radiation therapy (SBRT) was not recommended for the same reasons. Other options such as palliative chemotherapy and palliative radiotherapy were suggested as well as more aggressive treatment with concurrent chemoradiation. He consented to the last option and received 45-Gy in 25 fractions using VMAT with the last dose in September 2013. The gross tumor volume (GTV) was the large mass in the retroperitoneum just superior to the left renal vein between the SMA and the left adrenal gland. The clinical target volume (CTV) covered the other local regional lymph nodes to account for microscopic disease. And the planning target volume (PTV) was CTV plus 1 cm expansion to account for tumor movement, patient movement, daily setup error, etc. (Figure 4). The organs at risk (OARs) included the spinal cord, bone, liver, and both kidneys. All normal tissue tolerance dose constraints were met on the dose volume histogram (DVH) (Figure 5).

He had image-guided radiation therapy (IGRT) with daily cone beam CT (CBCT) guidance. CBCT was matched to the vertebral bodies with manual assessment of GTV and PTV. The treatment unit was Varian Clinac iX Linear Accelerator (Varian Medical Systems, Palo Alto, CA). We used 6MV photons and 720-degree arcs. Average machine time was only 5.92 minutes, from the time of starting CBCT to the completion of VMAT delivery. This helped with patient comfort and encouraged compliance.

**Figure 6:**
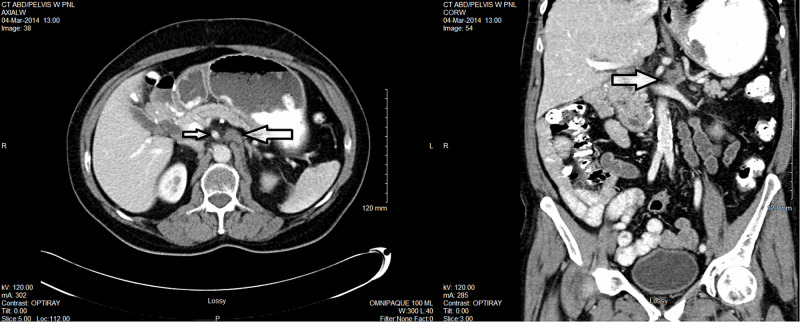
CT scan in March 2014 showing good PR. Note the residual mass (big arrow) and SMA (small arrow). PR, partial response; SMA, superior mesentery artery

**Figure 7:**
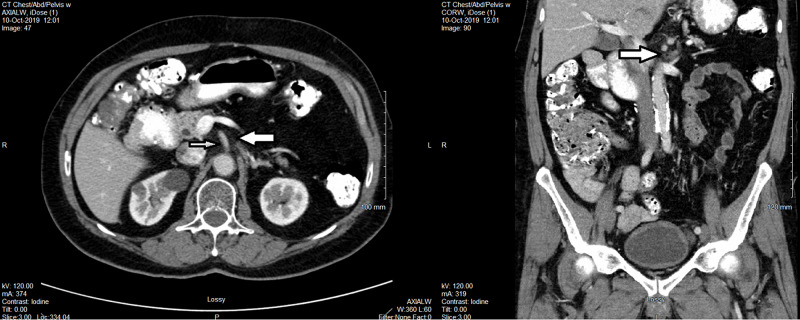
CT scan in October 2019 showing CR. Note the disappearance of residual mass (big arrow) beside SMA (small arrow). CR, complete response; SMA, superior mesentery artery

He also had concurrent chemotherapy with 5-FU and leucovorin for four days in week 1 and 5 of radiotherapy. He did not experience any significant acute toxicities from either radiation or chemotherapy. We achieved a good partial response on the first post-treatment follow-up CT scan (Figure 6). He was referred to two expert surgical oncologists in two different large academic cancer institutions. Both of them considered that it was too risky to do the retroperitoneal lymph node dissection, and not necessary especially when further CT scans showed complete response (CR) (Figure 7). He was simply followed with serial CT scans without any surgical intervention or further chemotherapy.

At the last visit in May 2020, he did not have significant late side effects from either chemotherapy or VMAT. His QoL was well maintained. There was no symptoms or CT imaging suggesting recurrent or metastatic cancer. Bone scan was also negative for bone metastases. He was considered cured seven years after salvage VMAT or eleven years after the initial diagnosis of SBA, but insisted to be followed on yearly basis.

## Discussion

SBC is very rare. In 2020, an estimated 11,110 new cases will occur in the USA and 1,700 patients will die of this disease [1]. SBA is even scarcer, accounting for 30%-50% of all SBCs. It has a poor prognosis with overall five‐year disease-specific survival (DSS) rate of 30.5% and median survival of 19.7 months, with 3.9% and 8.6 months in stage 4 metastatic disease, respectively [2]. The French study showed a similar outcome [8].

Adequate surgical resection including lymph node dissection is very important in prognosis. Overman et al. evaluated the impact of the number of positive lymph nodes (PLN) and total lymph nodes (TLN) on survival after curative resection of SBA. In total, 1,991 patients (n=1,216 with stage I/II and n=775 with stage III) were analyzed. Survival depended on the TLNs assessed and number of PLNs. The five-year DSS rate for patients with stage II disease was 44%, 69%, and 83% for 0 TLNs, 1-7 TLNs, and>7 TLNs, respectively. The five-year DSS for patients with stage III disease was associated with the number of PLNs (58% and 37% for <3 PLNs and ≥3 PLNs, respectively) [9].

Similarly, Ecker et al. analyzed 1,743 resected non-metastatic duodenal adenocarcinoma patients in the National Cancer Database (1998-2011). LN metastases were present in 865 (49.6%) patients. Increasing LN assessment was associated with an increased likelihood of nodal involvement (P = 0.008). In node-negative patients, increasing LN assessment was associated with a decreased risk of death, with the largest actuarial survival differences observed for ≥15 LN (hazard ratio [HR] 0.63, 95% confidence interval [CI] 0.48-0.82, P = 0.001) [10].

Duodenum is the main site of SBA occurrence (55%), followed by jejunum (18%) and ileum (13%). Metastatic SBA of the jejunum is rarely reported and the outcome is usually poor due to delay in diagnosis and limited curative treatment options. To date, only two established guidelines exist [8,11]. The treatment is usually palliative chemotherapy with first-line leucovorin, fluorouracil, and oxaliplatin (FOLFOX)/capecitabine and oxaliplatin (CAPOX) or second-line fluorouracil, leucovorin, irinotecan (FOLFIRI) and bevacizumab [3-6,8,11,12]. There is no evidence of improved survival after adjuvant chemotherapy versus surgery alone, regardless of lymph node status [13,14]. A large single-institution study with 491 patients showed poor overall survival with metastatic SBA and adjuvant chemotherapy was not associated with longer overall survival (p = 0.44). Complete resection provides the only means of cure, and the role for adjuvant therapy remains uncertain [15]. Some smaller retrospective studies suggested that lack of tumor resection, non-prescription of chemotherapy, liver metastasis, intra-abdominal lymph node metastasis, poor histological differentiation, and lympho-vascular invasion could be associated with poor survival outcomes [16,17]. Most studies did not recommend or even mention the option of radiation, even in their reported combined modality therapy group [8,14].

Tsushima et al. retrospectively reviewed clinical courses of 132 patients with unresectable or recurrent SBA who received chemotherapy at 41 institutions in Japan. Their results suggested that fluoropyrimidine-oxaliplatin combination therapy is the most promising first-line chemotherapy regimen [18]. This was further confirmed by a larger systemic review of eighteen studies (15 observational, 3 phase II) that investigated systemic therapy and six observational studies that investigated cytoreductive surgery (CRS) with intraperitoneal chemotherapy for SBA with peritoneal metastases [19]. Another multi-institutional registry study evaluated the outcome after CRS plus hyperthermic intraperitoneal chemotherapy for 152 patients with peritoneal metastases from SBA, but it was never established as standard treatment or tested in any randomized controlled trials [20].

Occasionally some patients do receive radiation therapy. They are usually treated primarily with palliative intent. The goal is to achieve better local control and QoL. Unfortunately, there is no significant improvement in survival in the past. Radiation oncologists and patients are reluctant to consider high dose radiotherapy due to the concern of severe side effects and worse QoL. Radiation is only reserved for symptom control, i.e. for pain, anemia due to tumor bleeding, or small bowel obstruction, etc. Adjuvant chemoradiation with capecitabine or infusional 5-FU was once considered an option for stage III duodenal cancer that was margin positive after resection, but there was no survival advantage comparing to chemotherapy alone. There were also less than 10 cases of neoadjuvant chemoradiation for unresectable or recurrent duodenal adenocarcinoma reported in the literature and no such report for SBA of the jejunum [11].

Our institution started VMAT program in 2010. It was intended to reduce toxicities to normal organs at risk (OAR) while maintaining the same or better local control of localized cancer versus 3-D conformal radiotherapy. The average machine time was reduced, from the time of starting on-board imaging (OBI) to the completion of treatment delivery, comparing to conventional intensity-modulated radiotherapy (IMRT). This helped with patient comfort and encouraged compliance. We also use IGRT with daily CBCT guidance, which offered the opportunity of manual assessment of GTV and PTV that was not possible with conventional OBI such as KV portal imaging. Fortunately, this achieved excellent local control and CR in this case.

It should be noted that we did not pursue standard palliative approach as recommended in the published guidelines due to the rarity of this disease and the limited evidence in the literature [8,11]. To our knowledge, this is the first report of recurrent adenocarcinoma of the jejunum with biopsy-proven retroperitoneal lymph node metastasis cured by chemoradiation with VMAT eleven years after the initial diagnosis.

## Conclusions

This case shows the benefit of offering aggressive chemoradiation for recurrent adenocarcinoma of the jejunum with retroperitoneal lymph node metastasis to achieve potential cure. VMAT with concurrent chemotherapy should be considered for highly select patients with unresectable oligo-metastasis and good performance status to limit toxicity and for the best QoL. Retroperitoneal lymph node dissection is not absolutely necessary after CR. Palliative systemic chemotherapy should not be started until all curative intent options have been discussed in tumor board meetings. If there are available curative options, then some of the patients might gain complete remission as seen in this rare case.
